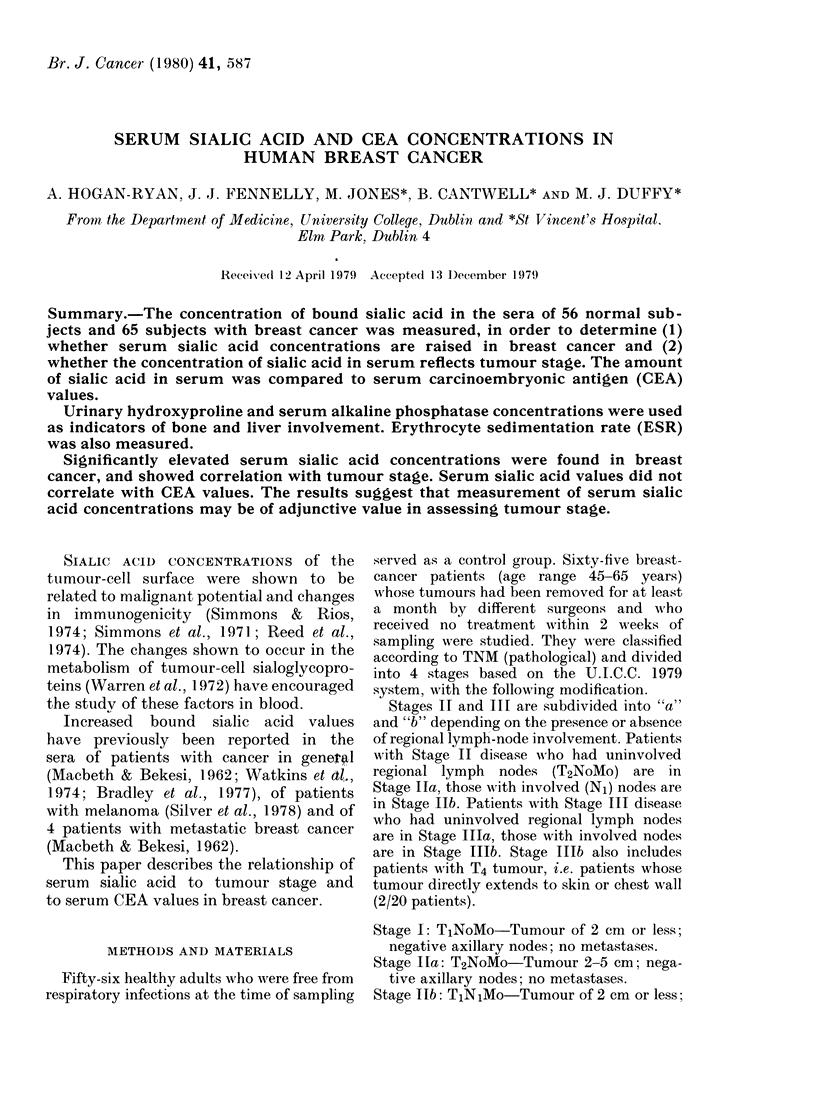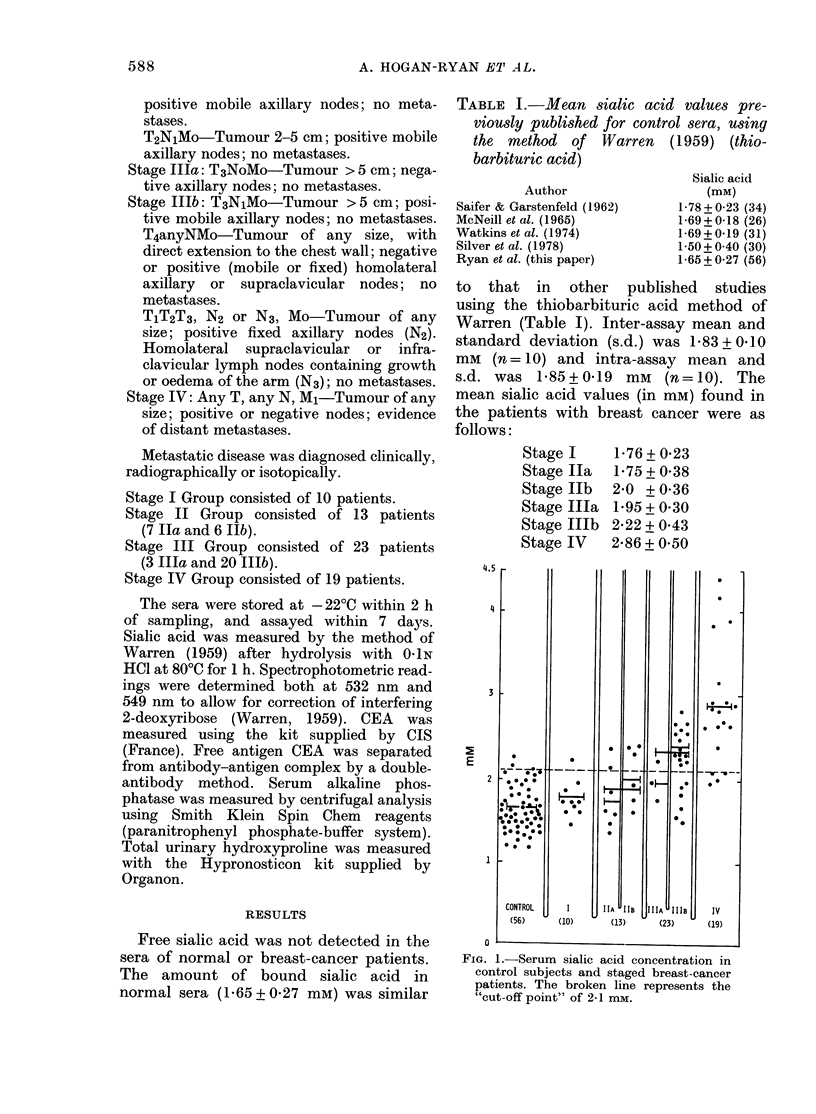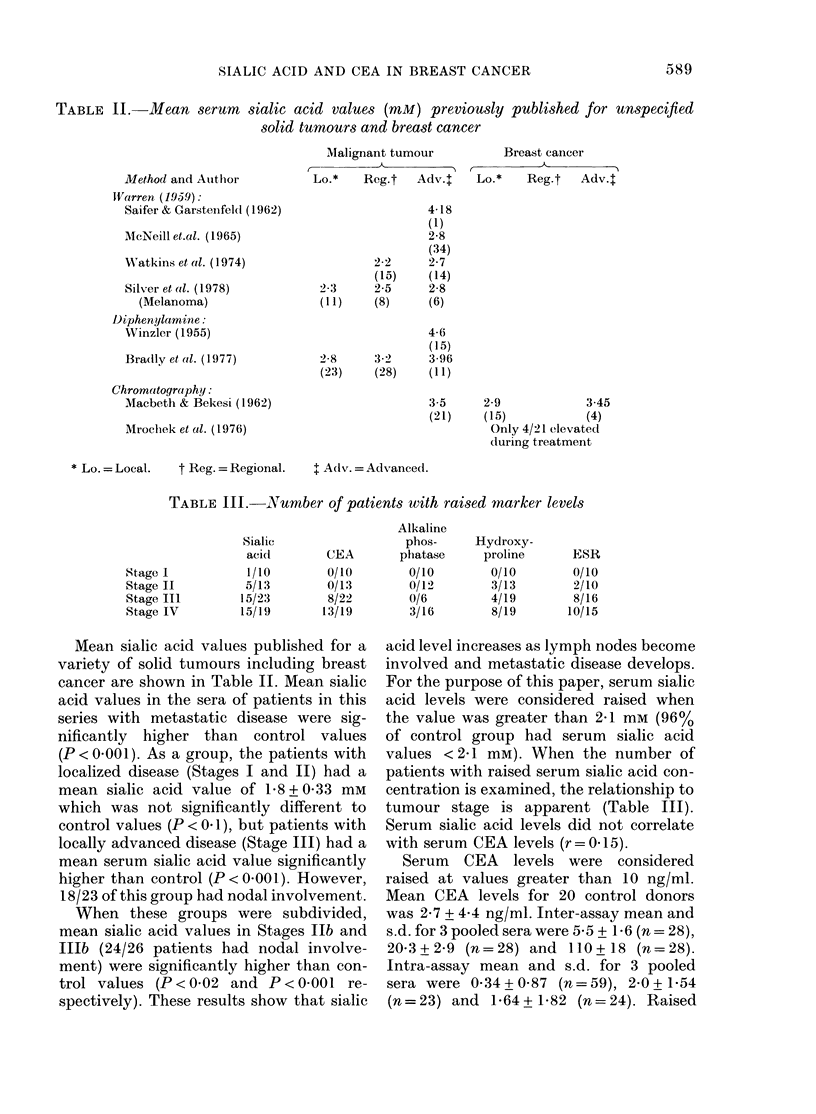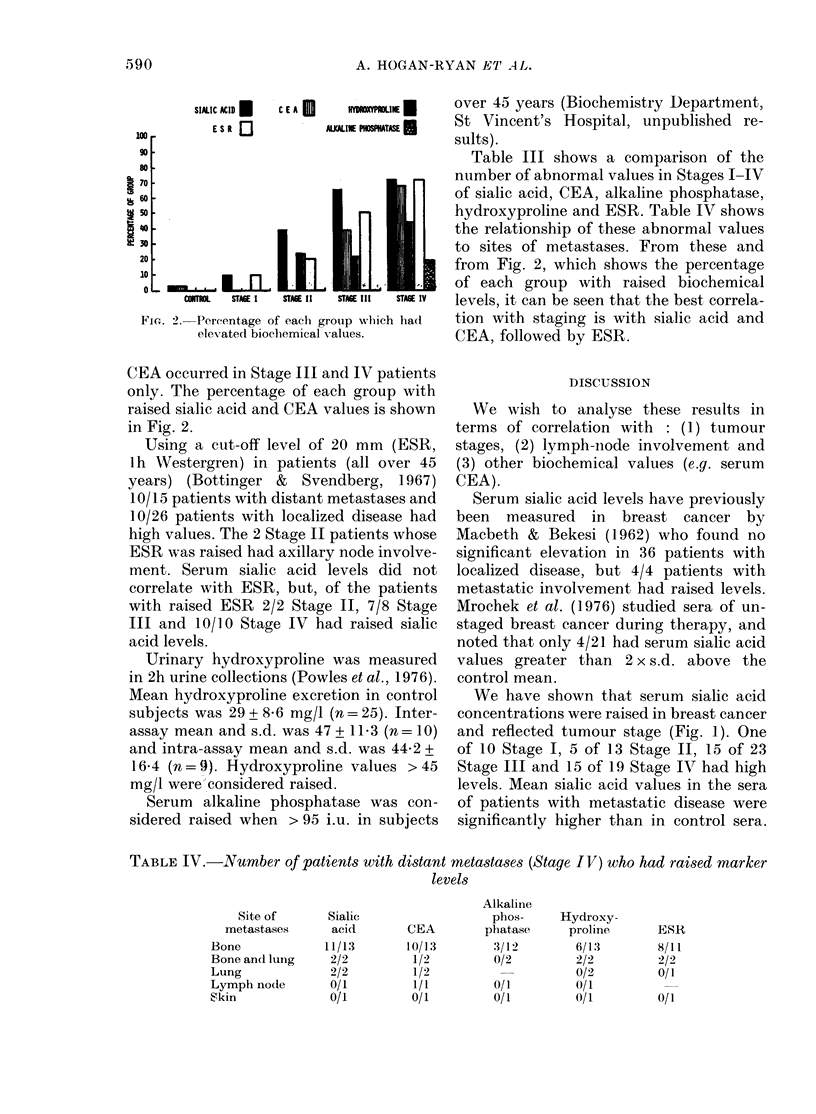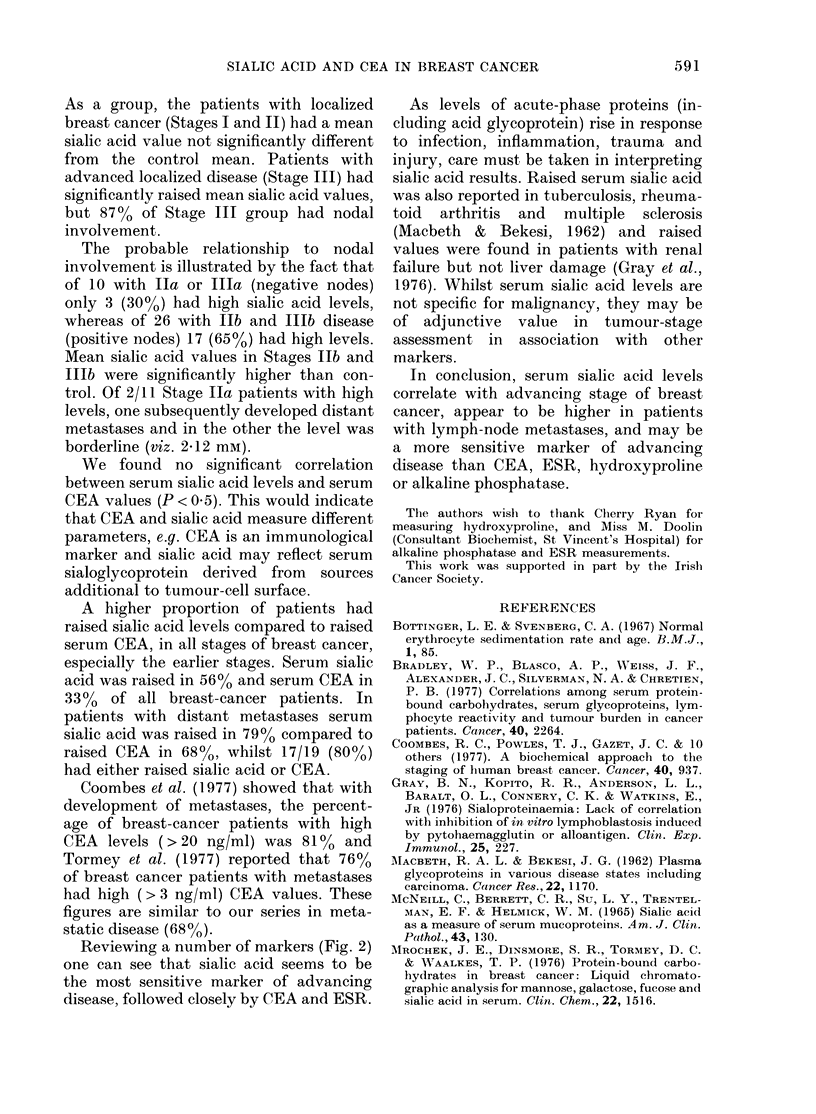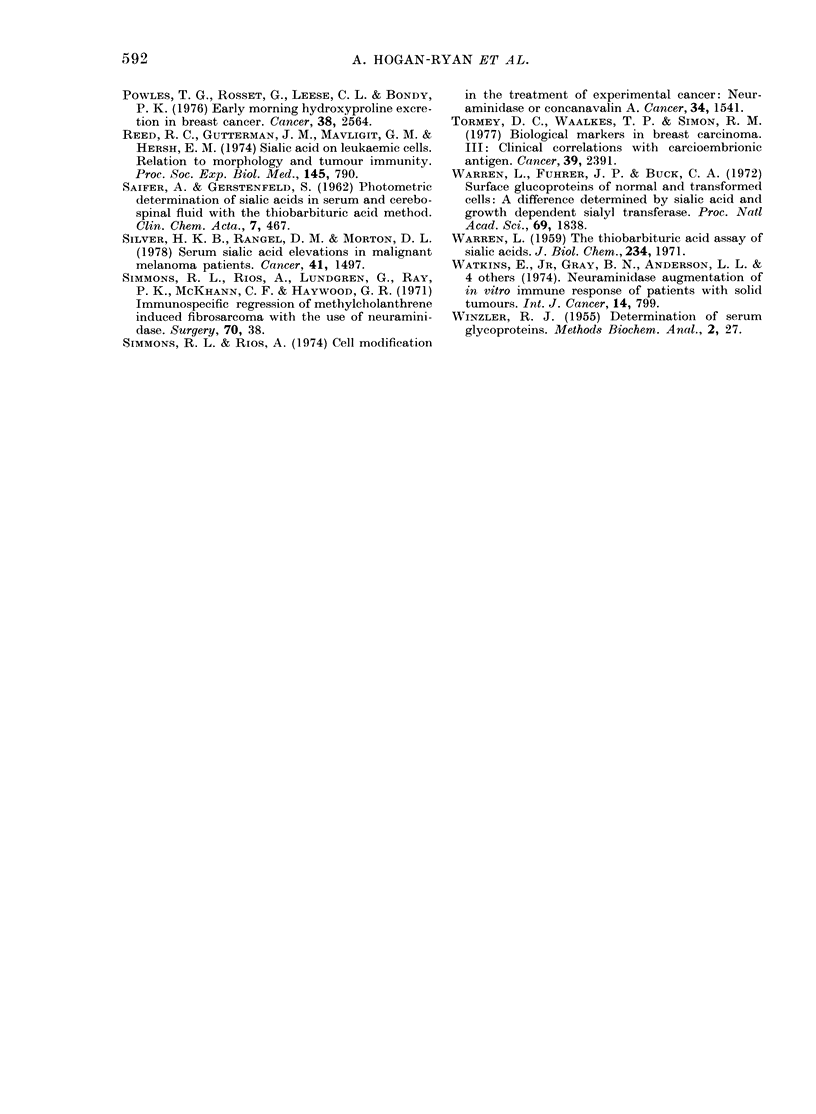# Serum sialic acid and CEA concentrations in human breast cancer.

**DOI:** 10.1038/bjc.1980.101

**Published:** 1980-04

**Authors:** A. Hogan-Ryan, J. J. Fennelly, M. Jones, B. Cantwell, M. J. Duffy

## Abstract

The concentration of bound sialic acid in the sera of 56 normal subjects and 65 subjects with breast cancer was measured, in order to determine (1) whether serum sialic acid concentrations are raised in breast cancer and (2) whether the concentration of sialic acid in serum reflects tumour stage. The amount of sialic acid in serum was compared to serum carcinoembryonic antigen (CEA) values. Urinary hydroxyproline and serum alkaline phosphatase concentrations were used as indicators of bone and liver involvement. Erythrocyte sedimentation rate (ESR) was also measured. Significantly elevated serum sialic acid concentrations were found in breast cancer, and showed correlation with tumour stage. Serum sialic acid values did not correlate with CEA values. The results suggest that measurement of serum sialic acid concentrations may be of adjunctive value in assessing tumour stage.


*Br. J. Cancer* (1980) **41**, 587

### SERUM SIALIC ACID AND CEA CONCENTRATIONS IN HUMAN BREAST CANCER

A. HOGAN-RYAN, J. J. FENNELLY, M. JONES*, B. CANTWELL* AND M. J. DUFFY*

*From the Department of Medicine, University College, Dublin and *St Vincent's Hospital, Elm Park, Dublin 4*

Received 12 April 1979 Accepted 13 December 1979

**Summary.—The concentration of bound sialic acid in the sera of 56 normal subjects and 65 subjects with breast cancer was measured, in order to determine (1) whether serum sialic acid concentrations are raised in breast cancer and (2) whether the concentration of sialic acid in serum reflects tumour stage. The amount of sialic acid in serum was compared to serum carcinoembryonic antigen (CEA) values.**

**Urinary hydroxyproline and serum alkaline phosphatase concentrations were used as indicators of bone and liver involvement. Erythrocyte sedimentation rate (ESR) was also measured.**

**Significantly elevated serum sialic acid concentrations were found in breast cancer, and showed correlation with tumour stage. Serum sialic acid values did not correlate with CEA values. The results suggest that measurement of serum sialic acid concentrations may be of adjunctive value in assessing tumour stage.**

SIALIC ACID CONCENTRATIONS of the tumour-cell surface were shown to be related to malignant potential and changes in immunogenicity (Simmons & Rios, 1974; Simmons *et al.*, 1971; Reed *et al.*, 1974). The changes shown to occur in the metabolism of tumour-cell sialoglycoproteins (Warren *et al.*, 1972) have encouraged the study of these factors in blood.

Increased bound sialic acid values have previously been reported in the sera of patients with cancer in general (Macbeth & Bekesi, 1962; Watkins *et al.*, 1974; Bradley *et al.*, 1977), of patients with melanoma (Silver *et al.*, 1978) and of 4 patients with metastatic breast cancer (Macbeth & Bekesi, 1962).

This paper describes the relationship of serum sialic acid to tumour stage and to serum CEA values in breast cancer.

### METHODS AND MATERIALS

Fifty-six healthy adults who were free from respiratory infections at the time of sampling served as a control group. Sixty-five breast-cancer patients (age range 45–65 years) whose tumours had been removed for at least a month by different surgeons and who received no treatment within 2 weeks of sampling were studied. They were classified according to TNM (pathological) and divided into 4 stages based on the U.I.C.C. 1979 system, with the following modification.

Stages II and III are subdivided into "*a*" and "*b*" depending on the presence or absence of regional lymph-node involvement. Patients with Stage II disease who had uninvolved regional lymph nodes ($T_2N_oM_o$) are in Stage II*a*, those with involved ($N_1$) nodes are in Stage II*b*. Patients with Stage III disease who had uninvolved regional lymph nodes are in Stage III*a*, those with involved nodes are in Stage III*b*. Stage III*b* also includes patients with $T_4$ tumour, *i.e.* patients whose tumour directly extends to skin or chest wall (2/20 patients).

Stage I: $T_1N_oM_o$—Tumour of 2 cm or less; negative axillary nodes; no metastases.

Stage II*a*: $T_2N_oM_o$—Tumour 2–5 cm; negative axillary nodes; no metastases.

Stage II*b*: $T_1N_1M_o$—Tumour of 2 cm or less; positive mobile axillary nodes; no metastases.

$T_2N_1M_0$—Tumour 2–5 cm; positive mobile axillary nodes; no metastases.

Stage III*a*: $T_3N_0M_0$—Tumour >5 cm; negative axillary nodes; no metastases.

Stage III*b*: $T_3N_1M_0$—Tumour >5 cm; positive mobile axillary nodes; no metastases.

$T_4$anyN$M_0$—Tumour of any size, with direct extension to the chest wall; negative or positive (mobile or fixed) homolateral axillary or supraclavicular nodes; no metastases.

$T_1T_2T_3$, $N_2$ or $N_3$, $M_0$—Tumour of any size; positive fixed axillary nodes ($N_2$). Homolateral supraclavicular or infraclavicular lymph nodes containing growth or oedema of the arm ($N_3$); no metastases.

Stage IV: Any T, any N, $M_1$—Tumour of any size; positive or negative nodes; evidence of distant metastases.

Metastatic disease was diagnosed clinically, radiographically or isotopically.

Stage I Group consisted of 10 patients.

Stage II Group consisted of 13 patients (7 II*a* and 6 II*b*).

Stage III Group consisted of 23 patients (3 III*a* and 20 III*b*).

Stage IV Group consisted of 19 patients.

The sera were stored at −22°C within 2 h of sampling, and assayed within 7 days. Sialic acid was measured by the method of Warren (1959) after hydrolysis with 0·1N HCl at 80°C for 1 h. Spectrophotometric readings were determined both at 532 nm and 549 nm to allow for correction of interfering 2-deoxyribose (Warren, 1959). CEA was measured using the kit supplied by CIS (France). Free antigen CEA was separated from antibody–antigen complex by a double-antibody method. Serum alkaline phosphatase was measured by centrifugal analysis using Smith Klein Spin Chem reagents (paranitrophenyl phosphate-buffer system). Total urinary hydroxyproline was measured with the Hypronosticon kit supplied by Organon.

### RESULTS

Free sialic acid was not detected in the sera of normal or breast-cancer patients. The amount of bound sialic acid in normal sera (1·65 ± 0·27 mM) was similar to that in other published studies using the thiobarbituric acid method of Warren (Table I). Inter-assay mean and standard deviation (s.d.) was 1·83 ± 0·10 mM ($n = 10$) and intra-assay mean and s.d. was 1·85 ± 0·19 mM ($n = 10$). The mean sialic acid values (in mM) found in the patients with breast cancer were as follows:

TABLE I.—*Mean sialic acid values previously published for control sera, using the method of Warren* (1959) (*thiobarbituric acid*)

| Author | Sialic acid (mM) |
|---|---|
| Saifer & Garstenfeld (1962) | 1·78 ± 0·23 (34) |
| McNeill *et al.* (1965) | 1·69 ± 0·18 (26) |
| Watkins *et al.* (1974) | 1·69 ± 0·19 (31) |
| Silver *et al.* (1978) | 1·50 ± 0·40 (30) |
| Ryan *et al.* (this paper) | 1·65 ± 0·27 (56) |

| | |
|---|---|
| Stage I | 1·76 ± 0·23 |
| Stage IIa | 1·75 ± 0·38 |
| Stage IIb | 2·0 ± 0·36 |
| Stage IIIa | 1·95 ± 0·30 |
| Stage IIIb | 2·22 ± 0·43 |
| Stage IV | 2·86 ± 0·50 |

FIG. 1.—Serum sialic acid concentration in control subjects and staged breast-cancer patients. The broken line represents the "cut-off point" of 2·1 mM.

TABLE II.—*Mean serum sialic acid values (mM) previously published for unspecified solid tumours and breast cancer*

| | Malignant tumour | | | Breast cancer | | |
|---|---|---|---|---|---|---|
| *Method* and Author | Lo.* | Reg.† | Adv.‡ | Lo.* | Reg.† | Adv.‡ |
| *Warren (1959):* | | | | | | |
| Saifer & Garstenfeld (1962) | | | 4·18 (1) | | | |
| McNeill *et.al.* (1965) | | | 2·8 (34) | | | |
| Watkins *et al.* (1974) | | 2·2 (15) | 2·7 (14) | | | |
| Silver *et al.* (1978) (Melanoma) | 2·3 (11) | 2·5 (8) | 2·8 (6) | | | |
| *Diphenylamine:* | | | | | | |
| Winzler (1955) | | | 4·6 (15) | | | |
| Bradly *et al.* (1977) | 2·8 (23) | 3·2 (28) | 3·96 (11) | | | |
| *Chromatography:* | | | | | | |
| Macbeth & Bekesi (1962) | | | 3·5 (21) | 2·9 (15) | | 3·45 (4) |
| Mrochek *et al.* (1976) | | | | Only 4/21 elevated during treatment | | |

* Lo. = Local. † Reg. = Regional. ‡ Adv. = Advanced.

TABLE III.—*Number of patients with raised marker levels*

| | Sialic acid | CEA | Alkaline phos-phatase | Hydroxy-proline | ESR |
|---|---|---|---|---|---|
| Stage I | 1/10 | 0/10 | 0/10 | 0/10 | 0/10 |
| Stage II | 5/13 | 0/13 | 0/12 | 3/13 | 2/10 |
| Stage III | 15/23 | 8/22 | 0/6 | 4/19 | 8/16 |
| Stage IV | 15/19 | 13/19 | 3/16 | 8/19 | 10/15 |

Mean sialic acid values published for a variety of solid tumours including breast cancer are shown in Table II. Mean sialic acid values in the sera of patients in this series with metastatic disease were significantly higher than control values ($P < 0{\cdot}001$). As a group, the patients with localized disease (Stages I and II) had a mean sialic acid value of $1{\cdot}8 \pm 0{\cdot}33$ mM which was not significantly different to control values ($P < 0{\cdot}1$), but patients with locally advanced disease (Stage III) had a mean serum sialic acid value significantly higher than control ($P < 0{\cdot}001$). However, 18/23 of this group had nodal involvement.

When these groups were subdivided, mean sialic acid values in Stages II*b* and III*b* (24/26 patients had nodal involvement) were significantly higher than control values ($P < 0{\cdot}02$ and $P < 0{\cdot}001$ respectively). These results show that sialic acid level increases as lymph nodes become involved and metastatic disease develops. For the purpose of this paper, serum sialic acid levels were considered raised when the value was greater than 2·1 mM (96% of control group had serum sialic acid values $< 2{\cdot}1$ mM). When the number of patients with raised serum sialic acid concentration is examined, the relationship to tumour stage is apparent (Table III). Serum sialic acid levels did not correlate with serum CEA levels ($r = 0{\cdot}15$).

Serum CEA levels were considered raised at values greater than 10 ng/ml. Mean CEA levels for 20 control donors was $2{\cdot}7 \pm 4{\cdot}4$ ng/ml. Inter-assay mean and s.d. for 3 pooled sera were $5{\cdot}5 \pm 1{\cdot}6$ ($n = 28$), $20{\cdot}3 \pm 2{\cdot}9$ ($n = 28$) and $110 \pm 18$ ($n = 28$). Intra-assay mean and s.d. for 3 pooled sera were $0{\cdot}34 \pm 0{\cdot}87$ ($n = 59$), $2{\cdot}0 \pm 1{\cdot}54$ ($n = 23$) and $1{\cdot}64 \pm 1{\cdot}82$ ($n = 24$). Raised

FIG. 2.—Percentage of each group which had elevated biochemical values.

CEA occurred in Stage III and IV patients only. The percentage of each group with raised sialic acid and CEA values is shown in Fig. 2.

Using a cut-off level of 20 mm (ESR, 1h Westergren) in patients (all over 45 years) (Bottinger & Svendberg, 1967) 10/15 patients with distant metastases and 10/26 patients with localized disease had high values. The 2 Stage II patients whose ESR was raised had axillary node involvement. Serum sialic acid levels did not correlate with ESR, but, of the patients with raised ESR 2/2 Stage II, 7/8 Stage III and 10/10 Stage IV had raised sialic acid levels.

Urinary hydroxyproline was measured in 2h urine collections (Powles *et al.*, 1976). Mean hydroxyproline excretion in control subjects was $29 \pm 8{\cdot}6$ mg/l ($n = 25$). Inter-assay mean and s.d. was $47 \pm 11{\cdot}3$ ($n = 10$) and intra-assay mean and s.d. was $44{\cdot}2 \pm 16{\cdot}4$ ($n = 9$). Hydroxyproline values $> 45$ mg/l were considered raised.

Serum alkaline phosphatase was considered raised when $> 95$ i.u. in subjects over 45 years (Biochemistry Department, St Vincent's Hospital, unpublished results).

Table III shows a comparison of the number of abnormal values in Stages I–IV of sialic acid, CEA, alkaline phosphatase, hydroxyproline and ESR. Table IV shows the relationship of these abnormal values to sites of metastases. From these and from Fig. 2, which shows the percentage of each group with raised biochemical levels, it can be seen that the best correlation with staging is with sialic acid and CEA, followed by ESR.

### DISCUSSION

We wish to analyse these results in terms of correlation with : (1) tumour stages, (2) lymph-node involvement and (3) other biochemical values (*e.g.* serum CEA).

Serum sialic acid levels have previously been measured in breast cancer by Macbeth & Bekesi (1962) who found no significant elevation in 36 patients with localized disease, but 4/4 patients with metastatic involvement had raised levels. Mrochek *et al.* (1976) studied sera of unstaged breast cancer during therapy, and noted that only 4/21 had serum sialic acid values greater than $2 \times$ s.d. above the control mean.

We have shown that serum sialic acid concentrations were raised in breast cancer and reflected tumour stage (Fig. 1). One of 10 Stage I, 5 of 13 Stage II, 15 of 23 Stage III and 15 of 19 Stage IV had high levels. Mean sialic acid values in the sera of patients with metastatic disease were significantly higher than in control sera.

TABLE IV.—*Number of patients with distant metastases (Stage IV) who had raised marker levels*

| Site of metastases | Sialic acid | CEA | Alkaline phosphatase | Hydroxyproline | ESR |
|---|---|---|---|---|---|
| Bone | 11/13 | 10/13 | 3/12 | 6/13 | 8/11 |
| Bone and lung | 2/2 | 1/2 | 0/2 | 2/2 | 2/2 |
| Lung | 2/2 | 1/2 | — | 0/2 | 0/1 |
| Lymph node | 0/1 | 1/1 | 0/1 | 0/1 | — |
| Skin | 0/1 | 0/1 | 0/1 | 0/1 | 0/1 |

As a group, the patients with localized breast cancer (Stages I and II) had a mean sialic acid value not significantly different from the control mean. Patients with advanced localized disease (Stage III) had significantly raised mean sialic acid values, but 87% of Stage III group had nodal involvement.

The probable relationship to nodal involvement is illustrated by the fact that of 10 with II*a* or III*a* (negative nodes) only 3 (30%) had high sialic acid levels, whereas of 26 with II*b* and III*b* disease (positive nodes) 17 (65%) had high levels. Mean sialic acid values in Stages II*b* and III*b* were significantly higher than control. Of 2/11 Stage II*a* patients with high levels, one subsequently developed distant metastases and in the other the level was borderline (*viz.* 2·12 mM).

We found no significant correlation between serum sialic acid levels and serum CEA values ($P < 0{\cdot}5$). This would indicate that CEA and sialic acid measure different parameters, *e.g.* CEA is an immunological marker and sialic acid may reflect serum sialoglycoprotein derived from sources additional to tumour-cell surface.

A higher proportion of patients had raised sialic acid levels compared to raised serum CEA, in all stages of breast cancer, especially the earlier stages. Serum sialic acid was raised in 56% and serum CEA in 33% of all breast-cancer patients. In patients with distant metastases serum sialic acid was raised in 79% compared to raised CEA in 68%, whilst 17/19 (80%) had either raised sialic acid or CEA.

Coombes *et al.* (1977) showed that with development of metastases, the percentage of breast-cancer patients with high CEA levels (> 20 ng/ml) was 81% and Tormey *et al.* (1977) reported that 76% of breast cancer patients with metastases had high (> 3 ng/ml) CEA values. These figures are similar to our series in metastatic disease (68%).

Reviewing a number of markers (Fig. 2) one can see that sialic acid seems to be the most sensitive marker of advancing disease, followed closely by CEA and ESR.

As levels of acute-phase proteins (including acid glycoprotein) rise in response to infection, inflammation, trauma and injury, care must be taken in interpreting sialic acid results. Raised serum sialic acid was also reported in tuberculosis, rheumatoid arthritis and multiple sclerosis (Macbeth & Bekesi, 1962) and raised values were found in patients with renal failure but not liver damage (Gray *et al.*, 1976). Whilst serum sialic acid levels are not specific for malignancy, they may be of adjunctive value in tumour-stage assessment in association with other markers.

In conclusion, serum sialic acid levels correlate with advancing stage of breast cancer, appear to be higher in patients with lymph-node metastases, and may be a more sensitive marker of advancing disease than CEA, ESR, hydroxyproline or alkaline phosphatase.

The authors wish to thank Cherry Ryan for measuring hydroxyproline, and Miss M. Doolin (Consultant Biochemist, St Vincent's Hospital) for alkaline phosphatase and ESR measurements.

This work was supported in part by the Irish Cancer Society.

### REFERENCES

BOTTINGER, L. E. & SVENBERG, C. A. (1967) Normal erythrocyte sedimentation rate and age. *B.M.J.*, **1**, 85.

BRADLEY, W. P., BLASCO, A. P., WEISS, J. F., ALEXANDER, J. C., SILVERMAN, N. A. & CHRETIEN, P. B. (1977) Correlations among serum protein-bound carbohydrates, serum glycoproteins, lymphocyte reactivity and tumour burden in cancer patients. *Cancer*, **40**, 2264.

COOMBES, R. C., POWLES, T. J., GAZET, J. C. & 10 others (1977). A biochemical approach to the staging of human breast cancer. *Cancer*, **40**, 937.

GRAY, B. N., KOPITO, R. R., ANDERSON, L. L., BARALT, O. L., CONNERY, C. K. & WATKINS, E., JR (1976) Sialoproteinaemia: Lack of correlation with inhibition of *in vitro* lymphoblastosis induced by pytohaemagglutin or alloantigen. *Clin. Exp. Immunol.*, **25**, 227.

MACBETH, R. A. L. & BEKESI, J. G. (1962) Plasma glycoproteins in various disease states including carcinoma. *Cancer Res.*, **22**, 1170.

MCNEILL, C., BERRETT, C. R., SU, L. Y., TRENTELMAN, E. F. & HELMICK, W. M. (1965) Sialic acid as a measure of serum mucoproteins. *Am. J. Clin. Pathol.*, **43**, 130.

MROCHEK, J. E., DINSMORE, S. R., TORMEY, D. C. & WAALKES, T. P. (1976) Protein-bound carbohydrates in breast cancer: Liquid chromatographic analysis for mannose, galactose, fucose and sialic acid in serum. *Clin. Chem.*, **22**, 1516.

POWLES, T. G., ROSSET, G., LEESE, C. L. & BONDY, P. K. (1976) Early morning hydroxyproline excretion in breast cancer. *Cancer*, **38**, 2564.

REED, R. C., GUTTERMAN, J. M., MAVLIGIT, G. M. & HERSH, E. M. (1974) Sialic acid on leukaemic cells. Relation to morphology and tumour immunity. *Proc. Soc. Exp. Biol. Med.*, **145**, 790.

SAIFER, A. & GERSTENFELD, S. (1962) Photometric determination of sialic acids in serum and cerebospinal fluid with the thiobarbituric acid method. *Clin. Chem. Acta.*, **7**, 467.

SILVER, H. K. B., RANGEL, D. M. & MORTON, D. L. (1978) Serum sialic acid elevations in malignant melanoma patients. *Cancer*, **41**, 1497.

SIMMONS, R. L., RIOS, A., LUNDGREN, G., RAY, P. K., MCKHANN, C. F. & HAYWOOD, G. R. (1971) Immunospecific regression of methylcholanthrene induced fibrosarcoma with the use of neuraminidase. *Surgery*, **70**, 38.

SIMMONS, R. L. & RIOS, A. (1974) Cell modification in the treatment of experimental cancer: Neuraminidase or concanavalin A. *Cancer*, **34**, 1541.

TORMEY, D. C., WAALKES, T. P. & SIMON, R. M. (1977) Biological markers in breast carcinoma. III: Clinical correlations with carcioembrionic antigen. *Cancer*, **39**, 2391.

WARREN, L., FUHRER, J. P. & BUCK, C. A. (1972) Surface glucoproteins of normal and transformed cells: A difference determined by sialic acid and growth dependent sialyl transferase. *Proc. Natl Acad. Sci.*, **69**, 1838.

WARREN, L. (1959) The thiobarbituric acid assay of sialic acids. *J. Biol. Chem.*, **234**, 1971.

WATKINS, E., JR, GRAY, B. N., ANDERSON, L. L. & 4 others (1974). Neuraminidase augmentation of *in vitro* immune response of patients with solid tumours. *Int. J. Cancer*, **14**, 799.

WINZLER, R. J. (1955) Determination of serum glycoproteins. *Methods Biochem. Anal.*, **2**, 27.